# Genetic affinities between endogamous and inbreeding populations of Uttar Pradesh

**DOI:** 10.1186/1471-2156-8-12

**Published:** 2007-04-07

**Authors:** Faisal Khan, Atul Kumar Pandey, Manorma Tripathi, Sudha Talwar, Prakash S Bisen, Minal Borkar, Suraksha Agrawal

**Affiliations:** 1 Department of Medical Genetics, Sanjay Gandhi Post Graduate Institute of Medical Sciences, Raebareli Road, Lucknow (UP) 226014 India; 2 Department of Biotechnology, J.C. Bose institute of Life Sciences, Bundelkhand University, Jhansi (UP) India

## Abstract

**Background:**

India has experienced several waves of migration since the Middle Paleolithic. It is believed that the initial demic movement into India was from Africa along the southern coastal route, approximately 60,000–85,000 years before present (ybp). It has also been reported that there were two other major colonization which included eastward diffusion of Neolithic farmers (Elamo Dravidians) from Middle East sometime between 10,000 and 7,000 ybp and a southern dispersal of Indo Europeans from Central Asia 3,000 ybp. Mongol entry during the thirteenth century A.D. as well as some possible minor incursions from South China 50,000 to 60,000 ybp may have also contributed to cultural, linguistic and genetic diversity in India. Therefore, the genetic affinity and relationship of Indians with other world populations and also within India are often contested. In the present study, we have attempted to offer a fresh and immaculate interpretation on the genetic relationships of different North Indian populations with other Indian and world populations.

**Results:**

We have first genotyped 20 tetra-nucleotide STR markers among 1800 north Indian samples of nine endogamous populations belonging to three different socio-cultural strata. Genetic distances (Nei's D_A _and Reynold's Fst) were calculated among the nine studied populations, Caucasians and East Asians. This analysis was based upon the allelic profile of 20 STR markers to assess the genetic similarity and differences of the north Indian populations. North Indians showed a stronger genetic relationship with the Europeans (D_A _0.0341 and F_st _0.0119) as compared to the Asians (D_A _0.1694 and F_st _– 0.0718). The upper caste Brahmins and Muslims were closest to Caucasians while middle caste populations were closer to Asians. Finally, three phylogenetic assessments based on two different NJ and ML phylogenetic methods and PC plot analysis were carried out using the same panel of 20 STR markers and 20 geo-ethnic populations. The three phylogenetic assessments revealed that north Indians are clustering with Caucasians.

**Conclusion:**

The genetic affinities of Indians and that of different caste groups towards Caucasians or East Asians is distributed in a cline where geographically north Indians and both upper caste and Muslim populations are genetically closer to the Caucasians.

## Background

Approximately, 60,000–85,000 years before present (ybp) the African exodus occurred and early modern humans got settled in South Asia. They moved further along the southern Asian coast to reach Southeast Asia, New Guinea, and Australia [[1,2], and [3]]. The exact entry date of modern humans into south Asia, which includes Bangladesh, Bhutan, India, Maldives, Nepal, Pakistan, and Sri Lanka, is uncertain. Various studies have been conducted in Indian specific to mtDNA or Y-chromosome. However, these macro haplogroups M and N lineages cannot explain the recent admixture from the neighboring region, which suggest that South Asian people are likely to have been settled in this region since the middle Paleolithic period [[4-8], and [9]]. Moreover, early Neolithic migration brought proto Dravidian speakers from the eastern horn of the Fertile Crescent. Approximately, 3,500 years ago the Indo-European speakers might have arrived and contributed to the Indian gene pool. The most recent conquerors from central Asia and the colonizers from Europe might have also added to this ethnic multiplicity.

It has been shown [10] that Indian populations exhibit high degree of genetic admixture and a greater genetic proximity with other world populations. Present day India is represented by a complex socio-cultural mosaic comprised of 20 major languages and approximately 750 dialects [11] constituted of 2000 castes and tribal groups [12]. The vast majority of these ethnic populations (at least 80%) are the Hindus, socially organized into castes and sub-castes [13]. Tribal groups comprise about 8 % of the total Indian population [14]. A third socio-religious group, the Muslims, which are represented by two sects, the Sunni and Shia, constitute approximately 12% of the total Indian population [15]. Several other minor religious and/or ethnic constituents include Jews, Christians, Buddhists and Sikhs among others. The relative frequencies of these groups vary regionally within the sub-continent [15].

It is believed that the Indian populations were derived from a small number of female founders, and the ethnic differentiation occurred subsequently through demographic expansions and geographic dispersal. This is further corroborated by the findings of shared haplogroup branches, without specific clustering of lineages in correlation with languages or the socio-cultural hierarchies of caste or tribal populations (except for Andaman tribal lineages) [4,11,16,17]. However, it has often been presumed that the tribes are the original inhabitants of India and responsible for most of the genetic pool of India. Linguistically, the tribal populations are classified into Austro-Asiatic, Dravidian, and Tibeto-Burman. Previous studies have revealed that the Austro-Asiatic tribes would have been the first in India [18]. These tribes might have entered India through the northwest, as they moved out of Africa following a path south of the Himalayas, while another ancestral group moved north, crossing the Himalayas, settling in southern China, and entering South Asia later from the northeast [14,17,19]. Basu et al. 2003 [14] studied mtDNA control region nucleotide diversity and have shown a high frequency (19%) of the ancient autochthonous haplogroup M2, and lack of the younger haplogroup M4, in these tribal groups. In contrast, recent analyses of Austro-Asiatic tribal populations showed a low frequency of the M2 haplogroup (~2%); considering this frequency, it would be difficult to claim that they were the earliest inhabitants of the Indian subcontinent [6]. In addition, mtDNA analysis of two southern Indian tribal groups in comparison to caste groups suggested that Indian caste and tribal populations share a common late Pleistocene maternal ancestry, while no genetic evidence substantiates the claim that the tribes were the earlier settlers, followed by caste groups derived from them [3,5,7,22]. Most of the studies have been conducted taking into consideration the mt DNA/Y binary markers. We have adopted a different approach by taking into consideration highly neutral markers (STRs) to study the genetic diversity among north Indians from the thickly populated state of India i.e. Uttar Pradesh.

To meet this objective, we genotyped 20 tetra-nucleotide STR markers among 1800 north Indian samples from nine endogamous populations belonging to three different socio-cultural strata. Three of the upper caste populations were Bhargavas, Chaturvedis and other Brahmins, while the middle caste populations were Kayastha, Mathurs, Rastogies and Vaish. Two Muslim populations were Shia and Sunni. Selection of the STR markers was based on the global survey carried out by Perez-Lezaun et al 1987 [23]. In order to provide a comprehensive picture of the genetic similarity and differences of the north Indian populations, various comparisons have been made.

## Results

### Allele frequency distribution

We have observed 176 alleles at 20 STRs. The allele number ranged from 7 to 9 at sixteen STR loci (THO1, TPO, FES, VWA, D4S243, DHFRP2, FGA, D7S820, D5S818, D11S2010, D2S1328, ACPP, D9S926, D13S1358, D14S306 and D18S848) while ≥ 10 alleles have been observed at the remaining four STRs i.e. D3S1358, D16S310, HPRT and F13A. Average number of observed alleles was 8.8. The maximum numbers of 164 alleles have been observed in Shia, and minimum in Vaish (146). All the STRs were in HWE. Locus wise average observed heterozygosity is shown in additional file 1.

### Estimation of Genetic distances

Pair wise genetic distances were calculated based on the allele frequencies of 20 STR markers. The distance matrices were generated [Table 1] and have shown almost similar picture by using Nei's and Renold's Fst method. All the nine populations were genetically closer to the European populations with combined Fst being 0.011 and combined D_A _being 0.034. Interestingly, all the nine population depicted relatively higher distances from Asian populations. Independently, the three upper caste Brahmin populations (Fst = 0.0116 and D_A _= 0.0332) were closest to the Europeans while four middle caste populations (Fst = 0.0167 and D_A _= 0.0482) were genetically more distant. The genetic distances with the Asian populations exhibited a totally different pattern where middle caste populations were least distant from Asians and upper caste Brahmins and Muslim sects were most distant [Table 1].

**Table 1:** Distance matrices (Nei's D_A _and Reynold's F_st _) calculated for nine north Indian populations with the Europeans and Asians based on the allele frequency data of 20 STR loci.

**Nei's D_A _genetic distance**		
	**Asia**	**Europe**
**North Indians**	0.1694	0.0341
**Upper caste**	0.1784	0.0332
**Middle caste**	0.1664	0.0482
**Muslims**	0.1893	0.0348
**South Indians (60 STR loci)**	0.0390	0.0450
**Fst Distance**		
**North Indians**	0.0718	0.0119
**Upper caste**	0.0746	0.0116
**Middle caste**	0.0710	0.0167
**Muslims**	0.0784	0.0122

### Phylogenetic reconstruction

#### Comparison with different world populations

**Figure 1:**
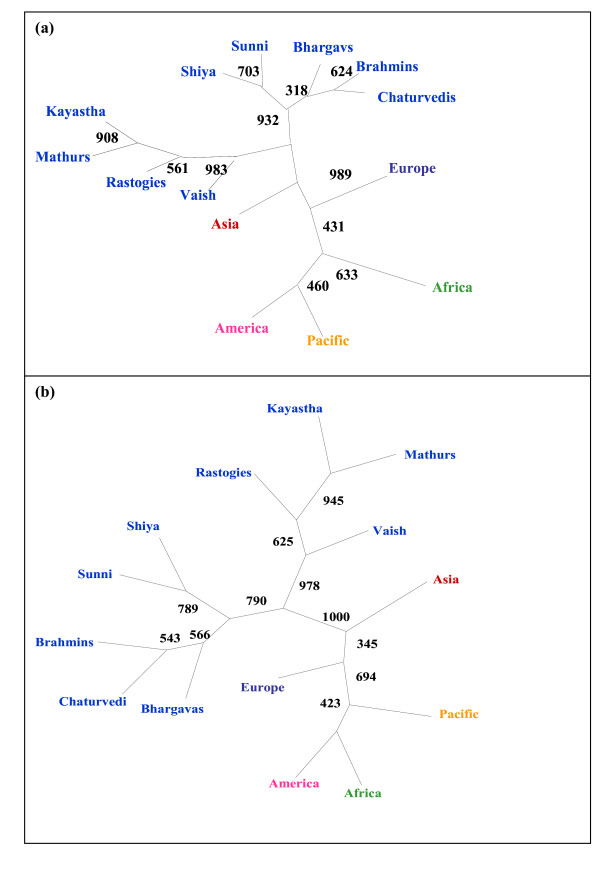
NJ and ML based Phylogenetic tree depicting clustering of the studied nine populations with five continental groups. (a) Maximum Likelihood (ML) tree with 1000 bootstrap replicates (b) Neighbor-joining (NJ) tree with 1000 bootstrap replicates.

**Figure 2:**
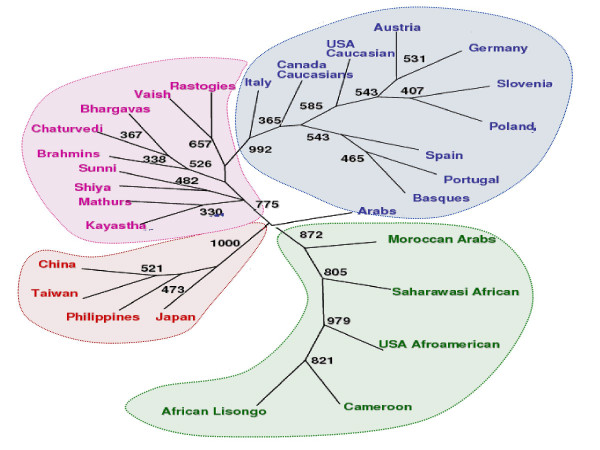
ML based Phylogenetic tree depicting clustering of the studied nine populations with 20 world populations. Blue: Caucasian populations; Green: African populations; Red: East Asia (oriental) populations; and Pink: North Indian populations (present study).

**Figure 3:**
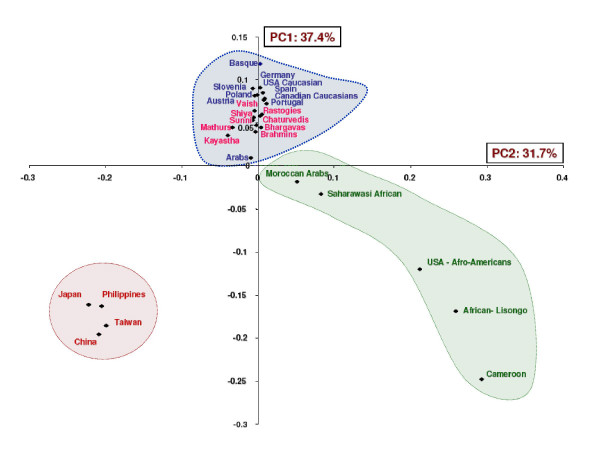
Phylogenetic reconstruction based on Principle component (PC-Plot) analysis. Blue: Caucasian populations; Green: African populations; Red: East Asia (oriental) populations; and Pink: North Indian populations (present study).

We have compared our populations with 16 world populations (Perez-Lezaun et al.1997 [23] which have been categorized into five major continents. Both NJ phylogram (Nei's D_A _genetic distance) and ML phylogram based on variance in allele frequency distribution on 20 STRs were analyzed [Figure 1a and 1b]. Our results revealed that middle caste populations (Kayastha, Mathurs, Vaish and Rastogies) are more similar to Asians while upper caste i.e. Brahmins, Bhargavas, and Chaturvedies are similar to Europeans. Interestingly Shias and Sunnis are closer to upper caste and, hence to Europeans. The ML phylogenetic tree and PC plot (Figure 2 and 3) generated from the allele frequency data of 29 populations (nine populations of the present study and twenty world populations) also depicted that the north Indian populations clearly clustered with the Caucasians.

#### Phylogenetic assessment of 35 Indian populations

**Figure 4:**
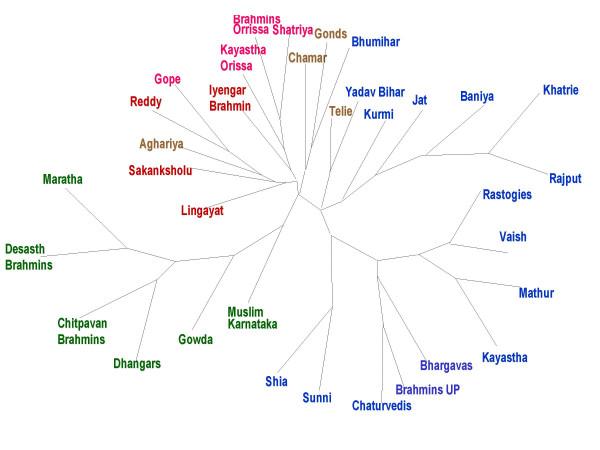
ML based Phylogenetic tree depicting clustering of the different Indian populations.

In order to provide a more comprehensive picture of the genetic similarity and differences existing between Indian populations, a population database comprising of 35 Indian populations was compiled. The criterion of selection was to cover the major geographical and ethnic groups from North, South, East, Central and Western India. Phylogenetic analysis was carried out in 35 populations. The phylogenetic assessments were made based on the allele frequency profile of 6 STR markers studied in all 35 populations. A phylogram was generated using maximum likelihood method [Figure 4]. The 6 STR loci included for the analysis are THO1, vWA, D5S818, D7S820, TPOX and D3S1358. All West Indian populations belonging to the state of Maharashtra have clustered in one group along with the two populations, Gowda and Muslims of the adjoining southern state of Karnataka. All West Indian populations belonging to the state of Maharashtra have clustered in one group along with the two populations, Gowda and Muslims of the adjoining southern state of Karnataka. All the populations analyzed in the present study, other populations of Uttar Pradesh and of adjoining state of Bihar formed a distinct cluster. The third cluster comprised of the populations of Eastern part of the country (Orissa) along with the south Indian upper caste Iyengar Brahmins. The fourth cluster was a conglomerate of populations from different regions. The nine studied populations of the present study clustered in the group of other north Indian populations but on a distinct branch. Another important aspect of the ML phylogram was the significantly longer branch length of north and West Indian populations, suggesting a high level of genetic diversity.

## Discussion

An elite historical, demographical and socio-cultural contour makes Indian populations a melting pot for the study of genetic variation and differentiation. On one hand, copious migratory events have created an extensive range of genetic diversity while inflexible and stern socio-cultural barriers have structured this diversity into different endogamous groups identified by the name of "castes". This term "Caste" is an assemblage of various socio-cultural customs; traditions and barriers that have created an abundant number of hierarchically arranged *'endogamous' *groups. This social hierarchy system is unique and exquisite because in the caste system, birth of an individual governs and decides most of the proceedings of the life including the choice of the mating partner. Marriage between partners of equal status is preferred, and reproduction in the caste system is largely endogamous [24].

In order to testify the hypothesis of social cleavage resulting into genetic structuring even in a confined geographical area, we have carried out various statistical analyses to determine the level of genetic differentiation between the studied populations. As mentioned earlier, the studied five groups belong to two different religious strata and practice highly restricted marital patterns. The level of population structuring triggered by the caste system further gets enhanced due to an additional level of endogamy called *'surname endogamy' *practiced mainly by the most stringent higher caste group [25].

We calculated the genetic distances and pair wise matrices using two different genetic distance calculations, Nei's DA, and Reynold's Fst. The distances were calculated from the European and Asian populations analyzed for the same set of 20 STR markers by Perez Lezaun et al [14]. Two genetic distance methods have been used to overcome the ascertainment bias resulted from hyper mutability of STR loci [17]. The estimation of genetic distances between Europeans and Proto-Asians (mainly East Asians) with north Indians at 20 STR markers clearly establishes strong genetic relationship of north Indians with Europeans as both DA (0.0341) and Fst (0.0119) reveled that north Indians are genetically more closer to the Europeans than Asians (DA 0.1694 and Fst – 0.0718). Another imperative finding was the differential pattern of genetic affinities of the three socio-cultural groups of north India with the Europeans. The affinity to Caucasians was proportionate to caste rank where upper caste Brahmins being genetically closest (DA = 0.0332 and Fst = 0.0116), followed by Muslims (DA = 0.0348 and Fst = 0.0122) while the middle caste populations were most distant from the Europeans (DA = 0.0482 and Fst = 0.0167) and closure to Asians.

In order to prove the phylogenetic accuracy of STR loci, a panel of only 10 autosomal STR loci has been used to infer phylogenetic relationships among well-defined geo-ethnic population groups and it has been observed that despite of the high allelic variability, STR loci are successful in reconstructing the accurate human phylogenies. To achieve this goal, a database of 22 geographically targeted and racially diverse set – population from forensic literature was compiled [33-41]. Both ML phylogram and PC-plot analysis were carried out and it was observed that basal cluster pattern of ML phylogram carries three geo-ethnic groups, indicating the role of genetic drift as a major force of evolution [Figure 2 and 3]. Both the trees have a longer African branch than any other group. Such a patristic separation was also visible in the PC-plot analysis [Figure 3]. The edge lengths displayed in these phylograms indicated the amount of evolutionary change along each branch. The scores next to the nodes characterize the number of bootstrap replicates (out of 1000) exhibiting these specific bifurcations. The African populations have been clustered into central (Cameroon and Lisongo) and North African (Moroccan Arabs and Saharawasi Africans) groups. Such clustering was also reported by Cavalli-Sforza and Feldman, (2003) and Underhill *et al.*, (2001) [28,29] on the basis of polymorphisms of 120 protein-coding genes and Y-chromosome binary haplogroup respectively. This sub clustering further strengthens the utility of STR loci in deciphering the accurate phylogenies, even within the same geographical region. Middle Eastern Arabs displayed a branch nearer to Caucasians and to some extent closer to Moroccan Arabs suggesting a strong Caucasian element along with African admixtures. This is indicative of the Demic expansion of the Middle East genes, agriculture innovations and languages into North West Africa [14], which is further supported by the near medial position of Arabs in the PC-plot. Recently, Y-chromosome SNP analysis by Al-Zahery *et al.*, 2003 [30] also revealed a similar pattern in other Middle Eastern populations. European branching pattern have resolved Basque, Spaniards and Portuguese in a separate cluster from that of German/Austrian branch. All 9 North Indian populations clustered on a separate branch emerging sharing the same node with the Caucasians in the ML phylogram.

We further compared our populations with other Indian and the world populations. On the basis of allele frequencies calculated at 6 STR loci in 35 Indian populations (including nine populations of the present study), we have deduced that the geographical barriers, and to some extent ethnic origin have been the major source of genetic structuring of Indian populations along with little effect of socio-cultural practices. Similar observations have been made by Kashyap *et al *2002 [27] in their report on 54 distinct Indian populations. Phylogenetic analysis of the 35 populations have yielded a geo-ethnically structured picture [Figure 3], where it has been seen that three well defined clusters comprising of north, west and east Indian populations were formed and in each of the geographic cluster, different caste and Muslim populations have clustered together. For instance, like Karnataka Muslims clustered with Desasth and Chitpavan Brahmins of Maharashtra and north Indian Muslims clustered with north Indian Brahmins. Interestingly, most of the populations of these three clusters have a Caucasian origin. The clustering points towards the possibility the West Eurasian or central Asian immigrants appointed themselves to predominantly belonged to castes of higher rank and both lower caste and tribes were least admixed with these groups [11].

**Figure 5:**
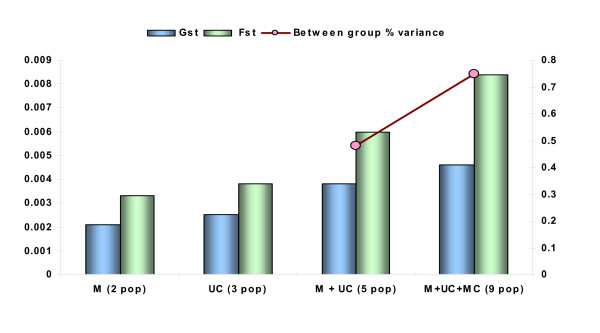
Comparison of Gst, Fst and AMOVA results among three socio-religious groups. M = Muslim populations (Shia and Sunni); UC = Upper caste populations (Bhargavas, Chaturvedis and Brahmins); MC = Middle caste populations (Kayastha, Mathurs, Rastogies and Vaish).

The results based on 20 STR markers have revealed that all the populations have similar allele frequency distribution which may be suggestive of a common ancestry or a continuous gene flow among these populations. We did Gst, Fst and AMOVA assessment for all the nine populations [Figure 5]. It was interesting to note that between group percentages variance got increased as the analysis was carried out between two (Muslim population), three (upper caste populations), five (upper caste) and all the nine populations. This finding revealed that the amount of genetic variation attributable to between populations in a socio-religious group is minimal as compared to variance between groups. Therefore, the three groups might differ from each other at the genetic level owing to the socio-cultural structuring. However, the genetic profile of all the nine populations included in the analysis exhibit extensive genetic overlap either due to the same common recent ancestor or due to the fact that the caste system is ~3000–4000 years old [26] and the time period is significantly small to create the genetic differentiation. Furthermore, the spread of Muslim population groups in India is ascribed to heavy admixture with local caste populations [13].

It has been proposed that the genetic make up of the Indian populations is an admixture of Caucasians and Asians. Bamshad et al. 2001 [11] studied 8 south Indian populations and deduced that they are genetically closer to Asian populations. The data generated from the present study has shown that the nine north Indian populations are genetically closer to the Caucasian than to the Asian populations. The phylogenetic reconstruction of these populations along with other world populations has also shown that all the nine north Indian populations have clustered with the European populations [Figure 1, 2, 3]. When genetic distance were calculated between Eurasians (West Eurasia, Middle East and Central Asia) and Proto-Asians (mainly East Asians) with north Indians at 20 STR and with south Indians at 60 STR loci [11], two different patterns of genetic distance were obtained [Table 2]. Geographically, genetic distance with the Eurasians increases from north to South India while that with proto-Asian populations decreases. The other observation was that when different caste groups of north India were compared with Eurasians and proto-Asian populations groups, the affinity to Eurasians was proportionate to caste rank while the upper caste and Muslims were more similar to Caucasians than middle caste populations.

**Table 2:** Pair wise genetic distance (D_A _) matrix for north and south Indians with Eurasian and Asians.

	**Eurasians**	**Asian**
**North Indians (20 STR loci)**	0.024	0.058
**South Indians (60 STR loci)**	0.045	0.039
**North Indian upper caste (20 STR loci)**	0.015	0.074
**North Indian middle caste (20 STR loci)**	0.030	0.042
**South Indian upper caste (60 STR loci)**	0.032	0.058
**South Indian middle caste (60 STR loci)**	0.057	0.032

## Conclusion

The phylogenetic analysis of nine endogamous/consanguineous populations of North India based on 20 STR loci have unwrapped the information about the genetic affinities of north Indians. Our results have demonstrated that intra-population differences were marginal still there was a definite pattern of genetic variation found in different populations. Finally, we observed genetic affinities of Indians and that of different caste groups towards Caucasians or East Asians which are differentially distributed where geographically north Indians and socially both upper caste and Muslim populations are genetically closer to the Caucasians. However, the genetic configuration of Indians is as complex hence more genetic data from the sub-continent is highly necessitated to unravel the intricacy of Indian genetic composition.

## Methods

### 1. Subjects

Present study is the first and one of the largest population based analysis (N = 1800) from this part of India. Moreover, we have taken into consideration a statistically significant sample size from all the caste populations. Total 200 samples have been collected from each of the nine populations i.e. Bhargavas, Chaturvedis, Brahmins, Kayastha, Mathurs, Rastogies, Vaish and Shia and Sunni Muslims (Uttar Pradesh). All the samples were adult with mean age of 38.8 ± 3.4 years and were residents of Uttar Pradesh since last three generations. Prior to the sample collection, regional addresses and detailed computerized lists of the populations have been prepared from different districts of Uttar Pradesh. Random numbers were generated with the help of computer and adult individuals living in different parts of Uttar Pradesh have been questioned about their ethnicity caste affiliations and surnames and the birthplaces of their parents as per the order of the random number. Only unrelated subjects were considered eligible to participate. The demographic profile and other ethnical and familial information were filled in a detailed Performa. Three-generation pedigree charts were prepared to assure un-relatedness in all the samples.

Whole blood was obtained by venipuncture and about 5 ml of blood was collected in EDTA vacutainer tubes after obtaining the informed consent from the subjects. The study was performed with the approval of the institutional ethical reviewing committee of Sanjay Gandhi Post Graduate Institute of Medical Sciences (SGPGIMS), Lucknow and the government of India.

### 2. DNA extraction

High molecular weight genomic DNA was extracted by salting out method using phenol-chloroform as described by Coomey et al. 1993 [31] and was purified by ethanol precipitation.

### 3. STR genotyping

A panel of 20 STR markers namely, FGA, D5S818, D7S820, D11S2010, D13S767, D9S926, D2S1328, D18S848, D14S306, D3S1358, ACPP, TPO, Tho1, VWA, FES, F13A1, D16S310, DHFRP2, HPRT and D4S243 were genotyped using PCR based locus specific amplification as mentioned earlier [23]. One of the primers for each of marker was labeled with a fluorochrome – VIC (D5S818, D13S767, D18S848, ACPP, VWA, D16S310 and D4S243), Ned (TPO, D9S926, FES, D14S306, D7S820 and DHFRP2) and 6-FAM (FGA, D3S1358, Tho1, D11S2010, D2S1328, F13A1 and HPRT). PCR amplification of all the markers was carried out by co-amplifying 15 STR loci in five multiplex PCR while remaining five markers were genotyped by single PCR. Size fractionation of the fluorochrome labeled amplicons was carried out by capillary electrophoresis in ABI-310 automated fragment size genetic analyzer (Applied Biosystems, USA). Size calling of the alleles at individual loci was done with GENESCAN v 3.1.2 software with the help of 500-ROX size standard (Applied Biosystems, USA). Once the size calling was completed, GENOTYPER v2.5.2 software was used to assign base pair size to each of the allele at respective STR marker and create the allelic profile of an individual for different markers.

### 4. Statistical analysis

Allele frequencies at each of the marker have been obtained by direct counting method from the observed number of alleles at a locus divided by total number of gametes. Deviation from the assumption of Hardy Weinberg equilibrium at genotypic frequencies for all markers in random north Indian population was estimated using Fischer's exact test based on 1000 Markov-Chain algorithm steps in Arlequin v2 software. Bonferroni correction to the p value was applied.

Two different genetic distances Nei's D_A _, and Reynold's F_st _based on the allele frequency distribution of 20 STR were calculated to assess the genetic relationship of north Indians with the Europeans and Asians. Both the distance matrices namely Nei's D_A _, and Reynold's F_st _were calculated using GENDIST option in PHYLIP v3.5c [32].

Phylogenetic analysis carried out for different populations by two enrooted radial phylograms, Neighbor Joining or NJ-phylogenetic Tree and Maximum Likelihood or ML-phylogenetic Tree. The NJ algorithm was used to construct the branching array from a matrix of genetic distances calculated from different distance matrices namely Nei's D_A _, and Reynold's F_st _using GENDIST option in PHYLIP v3.5c [32]. The option NEGHBOR in PHYLIP v3.5c was used to draw the phylogenetic trees from the distance matrices. Maximum likelihood (ML) algorithm was used for phylogenetic reconstruction using CONTML in PHYLIP v3.5c. In both NJ as well as ML method, statistical bootstrap involving 1000 replicates was carried out using SEQBOOT option of PHYLIP v3.5c. Finally, a consensus of 1000 trees (NJ and ML both) was drawn using CONSENSE option of PHYLIP v 3.5 [32].

## Authors' contributions

FK carried out laboratory experiments statistical analysis and drafted the manuscript. AKP, MT and ST were involved in sample collection and laboratory analysis. PSB helped in intrepretation of data and editing of the manuscript. MB helped in the statistical analysis. SA has conceptualized the paper provided important intellectual inputs in the preparation of the manuscript. All authors read and approved the final manuscript.

## Supplementary Material

Additional File 1 **Observed Heterozygosity at 20 STR markers among nine north Indian populations**. Locus wise average observed heterozygosity for 20 STR marker in nine populations (present study). Click here for file
