# Supplementary material for: Genetic affinities between endogamous and inbreeding populations of Uttar Pradesh
Source: BMC Genet. 2007 Apr 7;8:12. doi: 10.1186/1471-2156-8-12 (PMC1855350; doi:10.1186/1471-2156-8-12)
Supplement: Additional File 1 — Observed Heterozygosity at 20 STR markers among nine north Indian populations. Locus wise average observed heterozygosity for 20 STR marker in nine populations (present study). [file 1471-2156-8-12-S1.doc]

**Supplementary Table 1 (S1): Observed Heterozygosity at 20 STR markers among nine north Indian populations**

|  | **Bhargavas** | **Chaturvedis** | **Brahmins** | **Shia** | **Sunni** | **Rastogies** | **Vaish** | **Kayastha** | **Mathurs** |
| --- | --- | --- | --- | --- | --- | --- | --- | --- | --- |
| **HPRT** | 0.70 | 0.69 | 0.72 | 0.72 | 0.72 | 0.79 | 0.74 | 0.74 | 0.76 |
| **THO1** | 0.71 | 0.76 | 0.74 | 0.78 | 0.72 | 0.78 | 0.74 | 0.74 | 0.77 |
| **D3S1358** | 0.79 | 0.82 | 0.80 | 0.84 | 0.81 | 0.82 | 0.82 | 0.82 | 0.83 |
| **D16S310** | 0.78 | 0.77 | 0.75 | 0.75 | 0.74 | 0.77 | 0.78 | 0.79 | 0.82 |
| **F13A** | 0.6 | 0.65 | 0.66 | 0.79 | 0.70 | 0.76 | 0.69 | 0.78 | 0.76 |
| **TPO** | 0.74 | 0.75 | 0.74 | 0.76 | 0.78 | 0.76 | 0.78 | 0.76 | 0.79 |
| **FES** | 0.80 | 0.80 | 0.82 | 0.81 | 0.82 | 0.83 | 0.84 | 0.82 | 0.80 |
| **VWA** | 0.81 | 0.80 | 0.79 | 0.81 | 0.80 | 0.78 | 0.79 | 0.79 | 0.77 |
| **D4S243** | 0.73 | 0.72 | 0.76 | 0.75 | 0.73 | 0.77 | 0.78 | 0.77 | 0.68 |
| **DHFRP2** | 0.69 | 0.67 | 0.69 | 0.70 | 0.72 | 0.78 | 0.68 | 0.7 | 0.72 |
| **FGA** | 0.85 | 0.75 | 0.81 | 0.87 | 0.87 | 0.89 | 0.85 | 0.85 | 0.84 |
| **D7S820** | 0.79 | 0.76 | 0.73 | 0.80 | 0.73 | 0.76 | 0.76 | 0.81 | 0.84 |
| **D5S818** | 0.69 | 0.85 | 0.78 | 0.75 | 0.77 | 0.68 | 0.78 | 0.75 | 0.77 |
| **D11S2010** | 0.8 | 0.74 | 0.80 | 0.79 | 0.78 | 0.73 | 0.74 | 0.77 | 0.73 |
| **D2S1328** | 0.76 | 0.79 | 0.73 | 0.74 | 0.75 | 0.70 | 0.72 | 0.73 | 0.69 |
| **ACPP** | 0.73 | 0.69 | 0.74 | 0.8 | 0.68 | 0.68 | 0.62 | 0.59 | 0.63 |
| **D9S926** | 0.77 | 0.75 | 0.76 | 0.76 | 0.75 | 0.73 | 0.70 | 0.70 | 0.70 |
| **D13S767** | 0.67 | 0.65 | 0.59 | 0.63 | 0.68 | 0.67 | 0.68 | 0.65 | 0.66 |
| **D14S306** | 0.64 | 0.66 | 0.76 | 0.62 | 0.67 | 0.64 | 0.67 | 0.62 | 0.65 |
| **D18S848** | 0.66 | 0.66 | 0.66 | 0.61 | 0.66 | 0.65 | 0.68 | 0.64 | 0.64 |
| **Average** | **0.735** | **0.736** | **0.74** | **0.754** | **0.744** | **0.748** | **0.743** | **0.741** | **0.742** |
